# Astrophotography, a portal for engaging non-STEM majors in science

**DOI:** 10.1186/s40594-016-0053-0

**Published:** 2016-11-08

**Authors:** Mario A. De Leo–Winkler, Gabriela Canalizo, Gillian Wilson

**Affiliations:** Department of Physics and Astronomy, University of California, Riverside, 900 University Ave., Riverside, 92521 USA

**Keywords:** Education, Astronomy, Astrophotography

## Abstract

**Background:**

We report the results of an undergraduate course in astrophotography designed to engage non-STEM majors in the natural sciences to train future amateur astronomers and citizen scientists. Over 200,000 students enroll in introductory astronomy elective classes every year in the US alone, which will possibly be their only encounter with a natural science. The course relies on constructivist educational methods to teach data reduction and image processing methods while addressing mathematical anxiety. The goal of the course is to offer a positive experience in the natural sciences which has been linked to the education of potential citizen scientists and amateur astronomers, groups which historically have analyzed a great amount of data and have provided numerous discoveries.

**Results:**

Students enrolled in the course reported a higher understanding of data reduction, image processing, telescope and camera use. Most students were eager to take up astrophotography as a hobby, opening the path to become future citizen scientists and amateur astronomers. We found that the methods required to practice astrophotography create a natural constructivist teaching environment.

**Conclusions:**

The course can be reproduced elsewhere to teach non-science major students techniques in data reduction and image processing as positive experiences to introduce them to STEM fields in the future.

## Findings

### Introduction

Astronomy is a gateway into science. The concepts and questions it acknowledges create a sense of wonder and awe. Its biggest assets are the riveting images produced with the advent of space telescopes, adaptive optics, robotized missions, and the communications era, which according to (Rosenberg et al. 2014) “inspire us and promise answers to big questions” (p. 1). The images also “provide a gateway to increase scientific understanding, by clarifying how nature behaves and how the scientific method leads us to develop models of this behavior and then subject these models to rigorous tests” (National Research Council Astronomy and Astrophysics Survey Committee 2000). Over 200,000 non-science major students enroll in introductory astronomy elective classes every year in the U.S.A. alone, and for many college students, it is their only encounter with a natural science. Given its interdisciplinary links, astronomy is a particularly appropriate vehicle for teaching science to a wide audience.^1^ Citizen science in astronomy is a novel way to get the non-professional community involved in scientific research by fulfilling visual recognition tasks or reducing and analyzing specific data sets. These tasks would otherwise be computationally expensive or miss specific details (galactic morphology,^2^ variations in light curves,^3^ identification of craters,^4^ among others) when performed by computer algorithms specifically designed for the same tasks (Fortson et al. 2012). Participating citizens have expressed their main motivation is to contribute to original scientific research or are aware of the possibility of discovering something new (Raddick et al. 2013). Avocational scientific astronomers (Williams 2000) produce dozens of new discoveries every year, driven by a childhood or adolescent interest (Kannappan 2001) which was not pursued as a career opportunity. Astronomy, as described by Borne et al. (2009), is also an “innately engaging scientific context within which teachers can engage students in research investigations that make use of publicly accesible databases” (p. 5). With the overwhelming amounts of data that exist today, the ability to mine and reduce data is of utter importance in any field of research.

However, there are some factors that keep a large fraction of the general audience from participating, at any level, in the natural sciences. One of the main such factors is mathematical anxiety, which is a feeling of tension and fear that accompanies math-related activities (Richardson and Suinn 1972), which threatens achievement and participation in the natural sciences. Capable students avoid the study of mathematics, thereby eroding the resource base for STEM careers. Students in social sciences, humanities, and business taking pre-calculus or algebra courses during college present some of the highest mathematical anxiety levels (Hembree 1990). Increased confidence in the use of mathematical abilities can lead students to have a strong understanding of issues related to math literacy, including its importance to society, and help them grow into math-literate adults (Henrich and Lee 2011). Illustrated educational methods have a supportive function in learning factual knowledge. Lewalter (2003) mentions that “learning is enhanced when learners build referential connections between their separately developed verbal and visual representations of learning material” (p. 179). Astrophotography is an excellent vehicle for teaching visually and experimentally, which are aids encouraged by a constructivist method of education, proven to reduce mathematical anxiety in the classroom (Hughes 2016). The educational benefits of illustrative methods associated with astrophotography can also help students easily relate with complex astrophysical phenomenon.

Our astrophotography course was designed for undergraduate students mainly pursuing a major in social sciences, business, or arts. The course focused on giving students a general overview of modern astronomy, fostering the use of a scientific lexicon, and reviewing data reduction and image processing methods. The goals were to create students knowledgeable of the natural sciences and educate potential amateur astronomers and citizen scientists.

### Methods

#### Course

We created an astrophotography course which was presented in two different versions.

The first version was offered as part of two high-enrollment undergraduate courses, the “The Violent Universe” and “The History of the Universe,” designed for (but not limited to) students from non-science majors (social sciences, humanities, business, and arts). Both courses covered a very wide range of themes in astronomy and physics: from the Big Bang to the fate of the Universe, the laws of motion, the nature of light, and fundamental universal forces. Instructors made use of multiple resources to captivate their academically diverse and popular (up to 570 students) classes. Sessions were enriched by demonstrations, creative extra-credit projects, telescope observations of the sun and the night sky, visual aids, interactive questions, among other activities. To further enhance student involvement in science, instructors offered astrophotography as an extra-credit option for anyone taking any of the two aforementioned courses. This version was comprised of four sessions (one per week during 4 weeks) on the history, development, and subjects of (digital) photography, select subjects in astrophysics (celestial movement, celestial coordinates, optics), data reduction and image processing, one hands-on practice session with the astrophotography equipment, and one session on image acquisition. The enrollment was limited by space, and up to 40 students were allowed to sign up per quarter. This version was mostly informal, meaning that grades only enhanced their course grades with extra credit and homework/exams were not assigned. Students were graded based on their active participation during class, their ability to make informed decisions during the image acquisition session, and a final written report. This version of the course followed an NSF report suggesting that working in an informal natural science learning environment is a primary component for improving student involvement in STEM (Friedman 2008).

The second version was offered as a University Honors ignition seminar, specifically designed to “ignite” the minds of freshmen students by covering a variety of subjects from an interdisciplinary perspective. It was a four-unit course consisting of twenty-one 80-min sessions during 10 weeks, building upon the subjects from the first version of the astrophotography class, while also addressing the nature of the physical phenomena behind the cosmic objects to be photographed (which would otherwise be covered in the core course of the first version). There also were two practice sessions with the astrophotography instruments and three sessions for image acquisition (two night sessions—one out in the desert for best image quality—and one during daytime for solar image acquisition). Enrollment in ignition seminars was capped at 15 students by the university to ensure greater student-instructor interaction. Because this version is a full course, students were required to take quizzes.

**Fig. 1:**
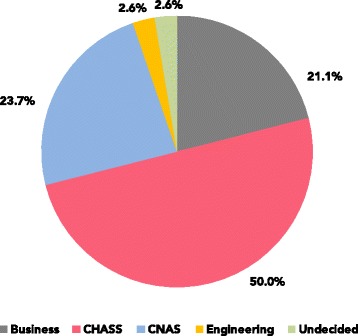
Course enrollment. Breakdown of the academic major enrollment of student population in the first version of the course, including students enrolled in CHASS (college of humanities, arts, and social sciences) and CNAS (college of natural and agricultural sciences)

Version 1 of the course was offered three times in 2015–2016 to a total of 98 students, of which 66 completed the course. Seventy-one percent of the students enrolled in the course were majoring in humanities, arts, social sciences, or business administration (henceforth *HASSBA*), while the rest were majoring in natural sciences or engineering (Fig. 1). Version 2 was given to 10 students (all HASSBA students), all of whom completed it.

#### Design

Due to the nature of the majors enrolled in both courses, we had to design it in such a way as to offer the core knowledge required to understand astrophysical phenomena, (digital) photography, the workings of a telescope and camera, celestial body movements, the nature of light, data reduction and image processing, without complex equations or complicated data/image processing methods and software. Therefore the course was divided into theoretical and practical sessions.

The theoretical sessions covered the following subjects: history of (astro)photography, the use of astrophotography, light pollution, the importance and nature of the celestial objects which are going to be photographed, exposure time, shutter speed, camera detector sensitivity (International Organization for Standardization, ISO), object tracking, star trails, focus, aperture, signal-to-noise ratio, camera sensors, types of telescope and mounts, focal length, magnification, celestial coordinates, and the importance and correction produced by dark/flat/bias images. Additionally, students were given a list of steps for best practices in astrophotography. This general guide ensured they knew how to first approach the available equipment, allowed them to make informed decisions on exposure times and sensitivity while using the camera, and prepared them to take as many images as possible to increase the signal-to-noise ratio in stacking procedures (which will be used afterwards), the procurement of bias, dark, and flat images.

The flow of theoretical sessions was interrupted midway by a practice session where the students had their first contact with the equipment. Participants were taught how to mount, balance, and align the tripods, telescopes, and cameras. The practice session was followed by an image acquisition session a few days afterwards.

In the image acquisition session, the telescopes and mounts were previously assembled by the instructor to focus the session mostly on taking pictures. The sessions took place during gray nights (when the half-moon is present in the night sky) to allow for simple astrophotography of our satellite. Students were required to take long-exposure photographs (defined by the instructors as having an exposure time of over 10 s) of previously assigned deep sky objects (nebulae, open clusters, or globular clusters) or planetary objects. The telescopes were either a Newtonian Celestron Advanced VX 6” or a Schmidt-Cassegrain Celestron Advanced VX 8”, both set atop an Advanced VX computerized and motorized equatorial mount. A Canon T5i DSLR (digital single-lens reflex) camera was attached to the telescope in prime focus through a T-mount adapter. Sessions started after astronomical twilight, to guarantee no additional light pollution. Students were guided and graded by the instructor, requiring them to follow their step-by-step guide for best practices in astrophotography and make decisions based on the actual observing conditions and the performance of the instruments on-site. The acquisition of images of the longest exposure time permitted by the quality of the tracking of the telescope mount or by light pollution was a requirement of the session. Students were questioned and graded jointly as a group during these sessions, allowing for joint decision-making and support by fellow classmates. Collaborative learning where groups work together to construct methods for approaching problems and get feedback on their ideas from their peers has been shown to increase confidence and reduce anxiety (Lavasani and Khandan 2011). Following the constructivist spirit of the course, students were expected to build upon previous knowledge from the theoretical sessions and experience from the practical session, group consensus, and experimentation with the equipment to make and support their decisions. Grades were assigned based on the criteria behind their decisions; decisions which result in higher quality images (less noise, more light, and enhanced detail) are assigned higher grades.

When the theoretical sessions continued, students used the acquired images to learn about data reduction and image processing, procedures and benefits of image stacking and alignment, and the difference between properties such as contrast and brightness. We used the Deep Sky Stacker^5^ software (DSS), due to its ease of use, high-quality results, free distribution, and its continuous testing and enhancement by the global astrophotographer community. Students were required to understand signal-to-noise ratios to establish a star-detection threshold which identifies common stars between images to align photographs of the same region in the sky. They also learned that an algorithm grades the quality of the images based on its blurriness and physical displacement from a specified reference image and that the user is allowed to select the percentage of images that are going to be stacked into a final composite based on this grade. Finally, they selected a stacking method, choosing between average or median values, to get the best results for the final image. The goal is to get as many images, from a single object, stacked to create a better signal-to-noise ratio, which will present richer detail and higher initial contrast. DSS natively works under Windows OS, but Mac OS users were given a DSS version, modified by the instructor, which works under the open Wine^6^ environment.

The resulting aligned and stacked images were then processed with GIMP,^7^ a free and open-source image manipulation program for Windows and Mac OS. There, students understood the use and manipulation of the histogram of an image, which is a graphic representation of its tonal distribution. By adjusting color curves, they changed and enhanced the contrast and brightness of the image. Image processing is a subjective exercise aligned with personal taste. Nevertheless, students were instructed to safeguard the maximum amount of stars, the highest level of detail, and increase the darkness of the image, understanding the workings of the program while experimenting.

The whole data reduction and image processing was first done with photographs of the celestial objects only, to be better acquainted with the procedure and the end result. The task is then repeated including dark, bias, and flat images. Students learned the usefulness of combining (bias/dark/flat) calibration images into a “master” image and their eventual subtraction or division to the stacked image. This process corrects problems in the telescope and camera and enhances the quality of the images. This allows the students to compare the benefits of using calibration images.

**Fig. 2:**
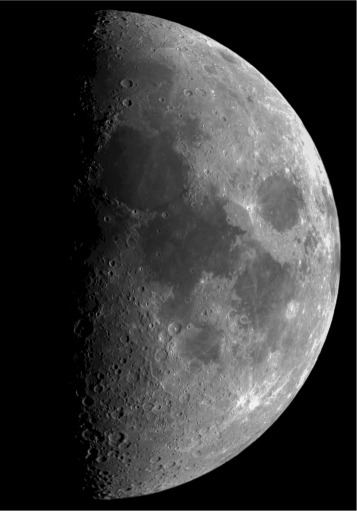
The moon. Final processed image of the moon taken with an 8-in. refracting telescope. The image is a seamless mosaic of two different images, which were properly focused and exposed during the image acquisition session. Afterwards, the image was reduced and processed to increase the level of detail and the contrast, which enhance the features on our natural satellite. Due to short exposure times and low noise levels, calibration images were not required for the moon

**Fig. 3:**
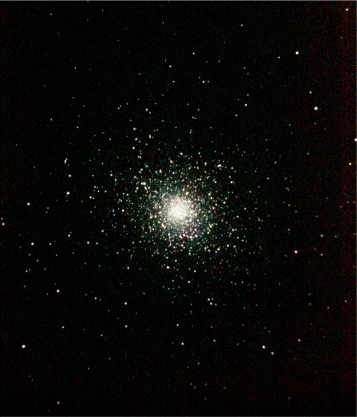
Globular cluster Messier 5. This cluster is a dense spherical group of hundreds of thousands of stars, captured with a 6-in. reflecting telescope. This final processed image allows the observer to detect the difference in the color of the stars, which is a measure of their temperature and mass

**Fig. 4:**
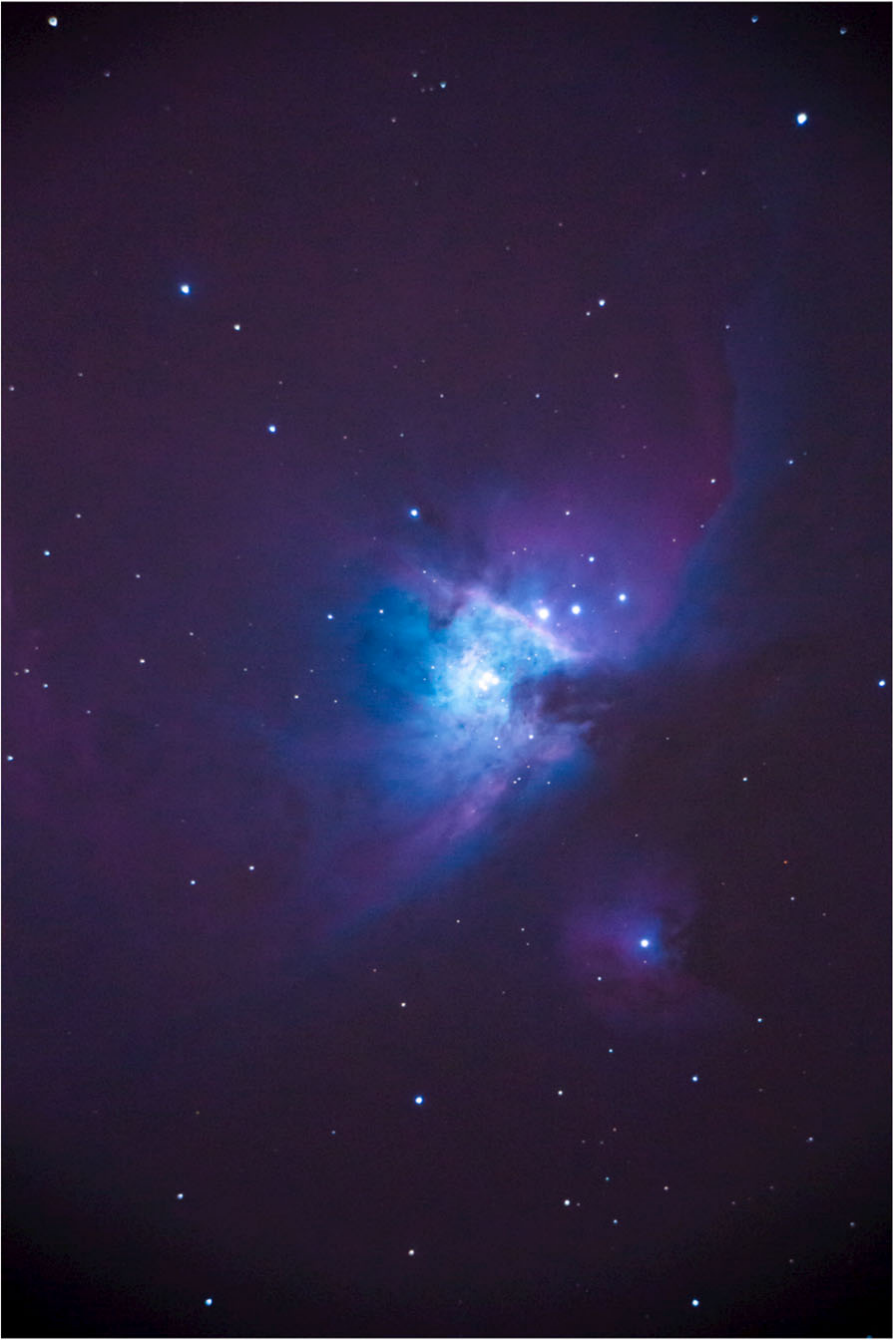
The Orion Nebula. This nebula is a star-forming region as observed with an 8-in. refracting telescope. The final processed image allows the observer to detect as much of the nebular gas as possible. Contrast allows for colors to stand out and provide information upon sight, *purple*/*red* indicates the presence of ionized hydrogen, *blue*/*green* the existence of doubly ionized oxygen, *white* is produced by starlight, and *irregular dark areas* are dust/gas clouds

Figures 2, 3, and 4 show the importance of precise telescope and camera calibration, focus, exposure times, image stacking, and use of calibration images, which allow subtle details to become visible. Color enhancement provides a wealth of information on the chemistry and physical properties (ionization, for example) of the cosmic objects.

For the final report, students were required to write about one of five subjects in layman’s terms. They were asked to write as if they were “explaining things to a relative or a friend.” This encouraged students to specifically report what they learned and not parrot the explanations that were given in class. Students were also expected to use their course notes, read, research, interpret, and describe their topic.

#### Mathematical anxiety

Our course needed to address mathematical anxiety, especially when 71 % of the attending students were majoring in fields with the highest reported anxiety levels.

Finlayson (2014) lists traditional educational delivery methods as the culprit for creating mathematical anxiety in the classroom. Exemplified by students receiving information passively, the teacher exercising power of authority, students working individually, and emphasis given to memorization and rote recitation rather than active concept learning, among other causes.

A constructivist teaching approach in the classroom has been reported to reduce mathematical anxiety (Finlayson 2014; Hughes 2016; NSES 1998). Posamentier et al. (2010) consider that when learning “emphasis must be placed on the process rather than the product” (p. 3). In constructivism, learners are actively involved in the process of meaning and knowledge construction, building upon previous knowledge. In the astrophotography courses, among many other precepts of constructivism, we highlighted the following:

- Learning through interaction
- Placing emphasis on understanding scientific concepts rather than memorizing facts and information
- Stimulating analysis and synthesis of data after defending conclusions
- Making the process itself an important part of the end product of learning
- Considering knowledge as dynamic and in constant change together with learning experiences
- Encouraging students to work in cooperative groups.

The design of the courses aimed to tackle mathematical anxiety in several ways. The use of the telescope and mounts required some basic knowledge of the equatorial coordinate system and the position of some guiding stars. Students were required to set, adjust, calibrate, and experiment with the equipment as a dynamic way to understand their working and handling based on the theoretical sessions. Environmental conditions (humidity, winds, clouds), equipment performance, and light pollution change during every image acquisition session, as well as from session to session. Students needed to assess these variables and decide on the best choices to obtain good-quality images. Simple mathematical formulas were required to calculate the magnification factor of the telescope, the exposure time of the camera, and the resulting signal-to-noise ratio of the final stacked image. Through on-site information and image previews provided by the equipment, students had more information to base their decisions to capture images. Student decisions were tested when an image was obtained; afterwards, they were required to discuss with their peers the quality of the resulting image and consider if changes needed to be made that would lead to a focused, well-exposed, and detailed image. They were required to explain to the instructor the reason behind their changes or support previous decisions, based on knowledge acquired during class. Data reduction can be performed in several ways to obtain similar results; we emphasized the understanding of the variables of the procedure to ensure all students obtained a similar result (darker images, enhanced color, increased signal-to-noise ratio). Image processing can be a subjective task, where personal taste for brightness, contrast, and color influence the end result; the instructor’s guidance helped students understand that there is no single standard for the end product but, instead, a wide variety exists that must fall within certain accepted parameters (good focus, dark, and detailed images). For the second version of the course, quizzes were written in such a form that answers were usually only a “true” or “false” statement; students needed to build upon previous knowledge and their understanding of concepts in order to arrive at the correct answer. Finally, a final written report was required, where students were requested to avoid technical terms and write a lay-termed personal understanding of cosmic phenomena, telescope/camera usage, data reduction, and image processing. Completeness, understanding, and concept abstraction of the subjects were graded.

We let the students work in groups, which allowed the creation of a personal bond and provided a structure of support, reducing anxiety caused by the classroom environment. We kept the topics concrete and, when possible, linked to practical and everyday analogies for physical phenomena. We used only the most essential of equations needed to determine the telescope’s magnifying power, the signal-to-noise ratio of stacked images, the collection area of telescopes, and the astronomical system of magnitudes. We made use of modern technologies such as computerized telescopes that are easy to set up and automatically track the position of celestial objects during the night, requiring just basic knowledge of coordinate systems and objects in the night sky. Amateur digital DSLR cameras, which provide a wealth of live information based on the user’s selected settings, were also used.

#### Evaluation

For the first version of the course, students were asked to complete an anonymous survey during the first session and an evaluation after the course had ended.

The survey during the first session had some questions which were asked again in the end. They allowed the instructors to assess some simple previous key knowledge of the course and were answered exclusively “yes” or “no.” The questions were as follows: “Do you know what image processing is?” and “Do you know what data processing is?”

The final evaluation was composed of questions and statements where the students agreed or disagreed within a scale from 1 (least favorable) to 5 (most favorable). The statements were in the style of “I have a better understanding of how telescopes work,” while questions were addressed similar to “Taking money issues aside, would you consider taking astrophotography as a hobby?”

This helped the instructors assess the level of satisfaction and newly acquired knowledge the students considered to have received during the course (understanding of telescope and camera workings, image processing methods, increased interest in astronomy, and evaluation of the theoretical and image acquisition sessions).

Students were also invited to provide additional comments, some of which are summarized in the “Statistics” section.

### Results

#### Statistics

We consider that the theoretical goals of the course were achieved, with a majority of the students reporting a higher understanding of how telescopes, cameras, and image processing work. On a scale from 1 (lowest grade) to 5 (highest grade), students reported better understanding (≥4) of the following: how cameras work (72.5 %), how telescopes work (65 %), and image processing methods (72 %).

**Fig. 5:**
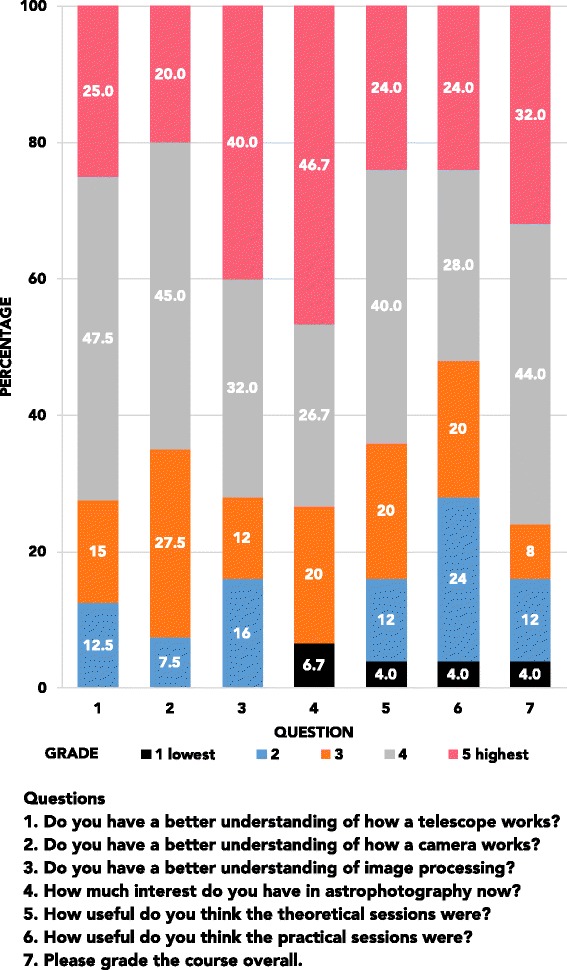
Results from evaluations. Summary of answers from the evaluations after the course was finished. Grades go from 1 (lowest) to 5 (highest). Questions: (1) Do you have a better understanding of how a telescope works?; (2) Do you have a better understanding of how a camera works?; (3) Do you have a better understanding of image processing?; (4) How much interest do you have in astrophotography now?; (5) How useful do you think theoretical sessions were?; (6) How useful do you think the practical sessions were?; (7) Please grade the course overall

A majority of students (69 %) graded the usefulness of theoretical sessions equal or above 4 (out of 5), compared to 62 % for the image acquisition sessions; the difference is probably due to the added complexity of the image acquisition session, where they are expected to make informed decisions based on previous knowledge. Students overwhelmingly (89 %) considered the course a very good/excellent (≥4) exercise in science. An increased interest in astronomy was also reported by 71 % of the students. The answers are shown in Fig. 5.

The first version of the course shows an attrition rate of around 23 %. Several factors can be held responsible: poor results in the base course, the non-binding nature of this extra-credit course, or the additional work load. The second version of the course, where students are bound by grades, presented no attrition, but the reasons for this are too diverse and beyond the scope of this paper.

When students were asked if, expenses aside, they would take up astrophotography as an extracurricular activity, 80 % answered affirmatively. This result supports our quest to create potential future amateur astronomers and citizen scientists.

Statistics on gender also produced interesting results. Studies have suggested that women are overlooked or socialized to dislike math and science in general (Geist and King 2008; Geist 2010). We can report a very high interest and lower attrition rate from female students. A total of 72 % of our students were women, and attrition rate was lower among female students (25.5 %) than among male students (45 %).

We present selected student comments on the course where the constructivist nature of astrophotography, and how it allows students to engage science in a positive setting, is clearly expressed:

- “Taking astrophotography was a great experience for me to really apply my learnings from the classroom into the real world.”
- “…[the course] presented material in an interactive and engaging way, and made information very easy to comprehend despite the occasional obscurity of the topic …”
- “…the practical aspects of the course […] made the course what it was, …[it] allowed us to practice and understand the theories explained in class …”
- “…instructions on the proper mounting, weighing, adjusting the telescope were confusing, but when we went out to the night session, it was easier to visualize.”

#### Experiences

The image acquisition sessions were performed on campus, at the outskirts of Riverside, California, which is embedded in an urban area. Light pollution presents a real problem; the Orion Nebula, and the brightest open and globular clusters, together with the moon, were easy targets, but galaxies and dimmer clusters lack detail. The urban jungle also creates additional atmospheric turbulence which is immediately recognizable when focusing a star or the moon. For this reason, we took students into the Mojave desert (Joshua Tree National Park), where conditions were much more favorable and the resulting images crisper and detailed. Equipment presented limitations also. Motorized and computerized equatorial mounts within our budget can only track celestial objects for up to 30 s in our best tests, forcing us to lower exposure times to avoid creating star trails, leaving most galaxies and nebulae out of our target list. The stacking of images will counter some of these pitfalls.

Finally, we caution that the effectivity of an astrophotography course to introduce data reduction and image processing to non-science fields through a constructivist perspective might rely heavily on local observing-site conditions. Several of our image acquisition sessions were canceled due to bad weather and later rescheduled, presenting an additional workload on the students. We then considered using archival astronomical images to perform data reduction and image processing. However, the comments in the students’ evaluations repeatedly emphasized that the image acquisition session was the best part of the course. The hands-on experience of obtaining celestial images as the result of an active decision making process created a very positive impact on the students. Additionally, image acquisition allows them to keep the usage rights of the images and use them as they best see fit. Therefore, locations with perpetual bad weather conditions for astronomy would probably benefit from photographing the moon and the sun instead. We consider these results alone to be a good example of the inherent appeal astrophotography has as a gateway into science.

### Conclusions

We used astrophotography as the vehicle to bring social sciences, humanities, business, and arts undergraduate students closer to data processing and image reduction, which are common tasks in the natural sciences and are becoming increasingly common in social sciences and business. Students also learned on subjects such as light, celestial movement, optics, calibration data, signal ratios, and common problems for astronomers such as light pollution and atmospheric turbulence.

The nature of astrophotography requires the employment of emerging technologies such as digital cameras, computerized telescopes, and data processing software; data reduction in astronomy permits students to follow multiple paths to obtain a good result; the subjective nature of image processing allows instructors to place additional emphasis on the process, not the method; the need of practical sessions to acquire photographs enhances the use of the material seen in the classroom. All aforementioned teaching tools, together with getting students to work in groups, follow a constructivist approach which has been proven to lower mathematical anxiety in the classroom (Finlayson 2014; Hughes 2016; NSES 1998; Posamentier et al. 2010). Therefore, we consider astrophotography to be a good tool to provide a positive scientific experience to undergraduate students majoring in social sciences, business, humanities, and the arts.

A high percentage of students reported a better understanding of how astronomical instruments work, the use of data reduction and image processing, considered the course a very good exercise in science, and would consider taking astrophotography as a hobby, which has been shown to later produce advocate scientific observers. Research shows that (among other reasons) interest and positive experiences in science earlier in life can help stimulate future individuals engaged in science (Kannappan 2001; Raddick et al. 2013). The incentive to create potential citizen scientists is of special interest, as they are currently pivotal in data mining, especially in areas where big data is handled. With a high percentage of our students majoring in social sciences, humanities, and arts, we have seeded positive background experiences in the natural sciences that would have otherwise not been available to them, making them learned citizens unafraid of approaching science.

## Endnotes

^1^ www.nap.edu/read/9839/chapter/7

^2^ https://www.galaxyzoo.org/

^3^ http://lcogt.net/agentexoplanet/

^4^ https://www.moonzoo.org/

^5^ http://deepskystacker.free.fr

^6^ http://www.winehq.org

^7^ http://www.gimp.org
